# Insect cell endocytosis of chikungunya virus adapted to Aedes albopictus, a mosquito recently introduced into southern France

**DOI:** 10.1186/1742-4690-9-S1-O8

**Published:** 2012-05-25

**Authors:** Christian Devaux, Eric Bernard, Bernard Gay, Nathalie Chazal, Laurence Briant

**Affiliations:** 1 Infectious Diseases at Cpbs (Centre d'Études d'Agents Pathogènes et Bio. Santé), Umr5236 Cnrs, Um1, Um2, Montpellier, France

## Introduction

Since the first isolation of chikungunya virus (CHIKV) more than 50 years ago in Eastern and Central Africa, CHIKV epidemics have been repeatedly recorded from various countries. The 2005-2006 outbreak in Reunion Island, was characterized by a genome microevolution in the E1 envelope glycoprotein gene (E1-A226V mutation) that enhances CHIKV fitness for Aedes Albopictus vector. More recently, CHIKV caused explosive outbreaks in India and propagated to temperate areas in Southern Europe, including France in 2010. Along with Aedes albopictus colonization of new geographical areas and climate change facilitating vector proliferation, the epidemic risk for "tropical infectious diseases" represents a real threat for naïve populations. It is therefore important to better understand the replication cycle of CHIKV in cells from Aedes albopictus.

## Materials and methods

CHIKV strains (the African reference strain of CHIKV 37997; the LR-OPY1 (E1-226V) variant isolated from Reunion Island and the LR-OPY1V226A bearing the reverse E1-V226A mutation were tested for replication in the C6/36 Ae. albopictus cell line. Experiments were performed to assess the role of clathrin and dynamin-dependent endocytic pathways implication of endosomal pH acidification and requirement for membrane cholesterol in CHIKV infection of mosquito cells.

## Result and conclusions

Our data indicate that CHIKV uses a clathrin-dependent, caveolae-independent pathway to infect Aedes albopictus cell cultures and requires membrane cholesterol as well as a low-pH environment for entry. These features, especially membrane cholesterol requirement, are modulated in some extent by the E1-A226V mutation. Altogether, our data provide the first information regarding the pathways used by CHIKV to infect Aedes albopictus cells and points the consequences of recent genome microevolution on these entry routes.

